# Plils: A Practical Indoor Localization System through Less Expensive Wireless Chips via Subregion Clustering

**DOI:** 10.3390/s18010205

**Published:** 2018-01-12

**Authors:** Xiaolong Li, Yifu Yang, Jun Cai, Yun Deng, Junfeng Yang, Xinmin Zhou, Lina Tan

**Affiliations:** 1 Key Laboratory of Hunan Province for New Retail Virtual Reality Technology, Hunan University of Commerce, Changsha 410205, China; yangyangyifu@yahoo.com (Y.Y.); b12100031@hnu.edu.cn (J.Y.); 2288@hnuc.edu.cn (X.Z.); lina@hnuc.edu.cn (L.T.); 2 Mobile E-business Collaborative Innovation Center of Hunan Province, Hunan University of Commerce, Changsha 410205, China; 3 Department of Electrical and Computer Engineering, University of Manitoba, Winnipeg, MB R3T 2N2, Canada; jun.cai@umanitoba.ca; 4 School of Information Engineering, Guilin Unversity of Technology, Guilin 541000, China; woshidengyun@sina.com

**Keywords:** wireless indoor localization system, subregion clustering, fingerprint, cheap communication chip

## Abstract

Reducing costs is a pragmatic method for promoting the widespread usage of indoor localization technology. Conventional indoor localization systems (ILSs) exploit relatively expensive wireless chips to measure received signal strength for positioning. Our work is based on a cheap and widely-used commercial off-the-shelf (COTS) wireless chip, i.e., the Nordic Semiconductor nRF24LE1, which has only several output power levels, and proposes a new power level based-ILS, called Plils. The localization procedure incorporates two phases: an offline training phase and an online localization phase. In the offline training phase, a self-organizing map (SOM) is utilized for dividing a target area into *k* subregions, wherein their grids in the same subregion have similar fingerprints. In the online localization phase, the support vector machine (SVM) and back propagation (BP) neural network methods are adopted to identify which subregion a tagged object is located in, and calculate its exact location, respectively. The reasonable value for *k* has been discussed as well. Our experiments show that Plils achieves 75 cm accuracy on average, and is robust to indoor obstacles.

## 1. Introduction

Wireless localization has become a prevalent service in recent years, and is categorized into two types: outdoor positioning and indoor positioning. Widely used outdoor positioning technologies include global navigation satellite systems (GNSSs), such as the global positioning system (GPS) and Beidou positioning systems. For indoor environments where satellite signals cannot commonly penetrate, indoor localization systems (ILSs) have become indispensable for localization services. Compared with other indoor localization technologies, such as vision analysis and motion sensors (accelerometers, compasses, gyroscopes), wireless indoor localization technologies can provide better coverage through whole buildings, and have the advantages of easy deployment and low maintenance. Hence, they can achieve both high accuracy and low cost [1]. At present, available wireless indoor localization systems generally rely on wireless infrastructures, including Zigbee, WiFi, Bluetooth, and radio frequency identification technology (RFID) [2].

In order to stimulate the development of wireless indoor localization systems, it is very important to decrease the hardware cost while maintaining functionality. We have investigated several widely used wireless communication chips for ILSs and listed their rough product prices in Table 1. Obviously, as a type of low-power low-cost commercial chip, nRF24LE1 from Nordic Semiconductor has the lowest cost. Thus, if this type of chip is able to achieve the specified localization precision, it will possess competitive superiority in terms of cost. However, during the process of designing and implementing nRF24LE1-based ILSs, it can be noted that there are limitations in nRF24LE1 that prevent this chip from being a potential solution for ILSs. For example, nRF24LE1 only supplies four output power levels and it has no capability for sensing and measuring the received signal strength. In addition, due to the impacts of multipath propagation effects and obstacles, the communication range of nodes equipped with nRF24LE1 communication chips is highly variable for different directions and environments. This can result in the values of the minimum received power level (MRPL) obtained by given receivers for different directions and different locations at the same transmission distance being very different.

Most existing works on wireless indoor localization focused on exploiting received signal strength. In [3], Chen et al. proposed a smartphone sensors-based pedestrian dead reckoning approach for ILSs, by leveraging various sensors and Bluetooth devices equipped on smartphones. Specifically, this approach utilized a magnetometer, gyroscope, and a rotation vector sensor to estimate walking length and walking direction, and the received signal strength (RSS) of Bluetooth signals to estimate walking distance. In [4], Kotaru et al. implemented a WiFi infrastructure-based indoor localization prototype, called SpotFi. SpotFi works on commercial off-the-shelf (COTS) WiFi access points (APs) to calculate the location of carry-on mobile objects based on information available in commercial chips, using the received signal strength indicator (RSSI) and channel state information (CSI). In [5], by taking into account the uncertainty of received RSSI values, Luo et al. introduced interval data to express the uncertainty of RSSI values, and proposed a mapping-based algorithm to calculate the locations of unknown nodes. In [6], the authors proposed a RFID positioning system, PinIt, which is suitable to be deployed in environments with a rich multipath and non-line-of-sight (NLOS). Assuming that nearby RFID tags exhibited similar multipath profiles, PinIt acquired the multipath profile of the target tag via synthetic aperture radar and adopted the dynamic time warping technique to find the nearest anchor tag. Spatial-temporal phase profiling (STPP) in [7] further improved localization accuracy. By moving the reader while continuously interrogating a set of tags, spatial-temporal phase profiles of these tags could be acquired, and then the information of phase profiles was leveraged to enable the relative RFID indoor localization. In [8], Soltani et al. employed the idea of clustering and proposed a localization algorithm, called CMTL+, which is suitable for movable objects attached with RFID tags. In CMTL+, by using *k*-nearest neighbor (*k*-NN) algorithm, some reference tags were clustered according to signal pattern similarity. A virtual reference tag (VRT) was created by an irregular bilinear interpolation method to estimate the RSSI values of each VRT based on those of real reference tags. Then, the BP network estimated the coordinates of an unknown target based on the RSSI information between itself and all reference tags in the cluster. However, all these works implemented positioning based on RSS or phase values, so they are not feasible for chips without the ability to measure these values.

In literature, few works are dedicated to power level-based RFID indoor localization, except LANDMARC in [9]. In the setting of LANDMARC, every reader supplied eight different power levels and recorded the MRPLs of unknown tracking tags, wherein the MRPL of one tag was defined as the minimum transmitted power level so that the reader could correctly decode data frames emitted from the tag. For a given tracking tag, a power level vector was set up consisting of the MRPLs from itself to different readers. By comparing the power level vector of the tag with those of anchor tags, the top *t* most closely matching anchor tags were able to be identified. Then, different weights associated with these *t* anchor tags were calculated and the tracking tag’s location could be roughly derived based on both the derived weights and the known locations of anchor tags.

Motivated by the growing interest in using commercial products or chips to achieve ILSs, in this paper, a power level-based indoor localization scheme using nRF24LE1 chips, called Plils, is proposed, which is similar to PinIt [6] and STPP [7], both of which utilize COTS RFID readers and tags. The selection of nRF24LE1 is based on its advantages in terms of its low cost, wide range of wireless application products, and long battery life time. Our goal is to provide a complete power level-based ILS solution with optimal performance in hardware cost and localization accuracy. Considering that fingerprinting-based localization strategy is inherently robust to the impact of obstacles, this paper adopts a fingerprint matching idea for localization, and proposes a power level-based indoor localization approach specified for nRF24LE1.

There are many research works on utilizing RSS fingerprinting to achieve localizations, especially in rich-obstacle environments. In [10], Stella et al. presented three typical kinds of localization determination techniques based on radio frequency fingerprinting, and derived the Cramer–Rao lower bound (CRLB) of localization accuracy for RSS measurements. Callaghan et al. [11] exploited pairwise cross correlations of the signals received at the nodes to estimate distances between them. Based on this, by using multidimensional scaling methods, an approximate map of locations was constructed for radio frequency localization. In [12], the authors evaluated performance of bluetooth low energy (BLE) localization based on RSS fingerprinting by mapping propagation modes in indoor scenarios. Our earlier proposed wireless ILS in [13] exploited the Markov chains to remove the fluctuation in MRPLs in operation and utilized a 1-nearest neighbor (1-NN) classifier to find the most similar mapping to estimate the locations of unknown nodes.

In contrast to all prior works in fingerprint-based localization, our proposed approach exploits the self-organizing map (SOM) technique to first divide the target region into several small subregions based on the similarity of signal fingerprints. After that, the support vector machine (SVM) is used to identify the tagging object’s subregion from all subregions. Finally, the evaluation results demonstrate the viability of both SOM and SVM, which can correctly identify the subregion a tagged object is located in and significantly reduce the search space for pinpointing its nearest anchor node.

The main contributions of this paper are summarized as follows. Firstly, a median location accuracy of 75 cm can be achieved by our practically proposed indoor localization system (Plils) built on four power level-based commodity chips. There are many practical applications for the proposed Plils. Some examples include forklift localization in intelligent warehouse and personnel positioning in the inspection system. Secondly, in contrast to prior works [5,6,7,9], which usually adopt a global matching idea to identify the nearest reference node for mobile tagging objects, Plils proposes a new matching framework, where subregion clustering enables assigning the current fingerprint vectors of mobile objects to their located subregions, so that it can avoid making large errors in localization. Thirdly, we analyze the relationship between the range λ of regions that mobile objects might be located in, and the number of subregions *k* to help choose the appropriate value of *k*, which has not been addressed before in literature.

The remainder of this paper is organized as follows. In Section 2, we present the basic transmission profile of nRF24l01 chips, and describe the main framework of our Plils system. In Section 3, we elaborate the implementation procedure of both offline training and online localization phases in detail. Section 4 presents the experimental results of Plils. Section 5 concludes this paper.

## 2. The Framework of Plils

**Figure 1:**
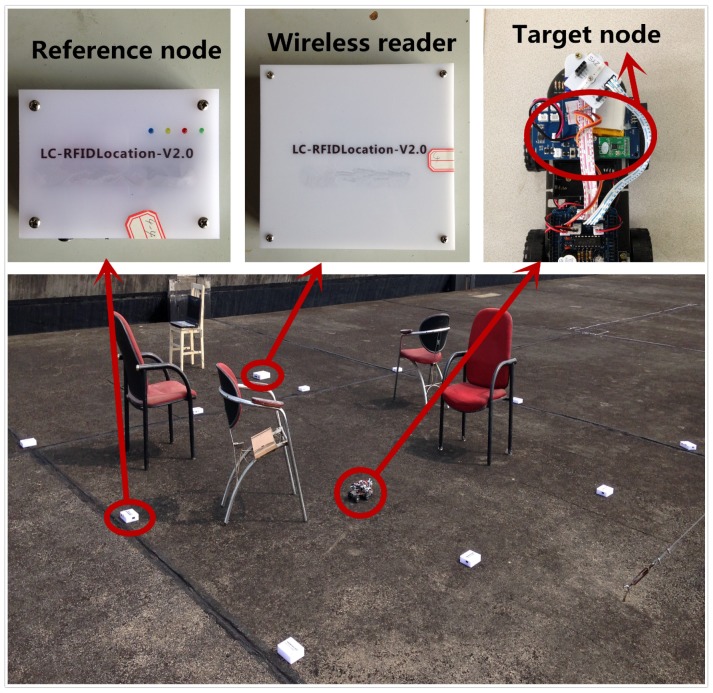
A photo of the layout of the experimental environment. RFID: radio frequency identification technology.

Similar to the conventional fingerprint-based wireless ILS, the proposed Plils consists of the following basic components: one wireless reader, stationary reference nodes, and mobile target nodes, as illustrated in Figure 1. A target node is associated with a moving object required to be localized, such as equipment, retail products, and machine devices. Compared with other ILSs, Plils has apparent distinctions in many aspects. First, Plils only requires one reader, whereas LANDMARC needs multiple RFID readers to achieve accurate localization. Besides, most existing ILSs rely on chips which are able to measure continuous received signal strength, whereas for Plils, all nodes are built upon a type of widely-used commercial system on a chip (SoC), nRF24LE1, which only supplies four discrete power levels, i.e., −18 dBm, −12 dBm, −6 dBm, and 0 dBm [14]. According to empirical measurement results, under the condition that no external antenna is added to nodes, the corresponding transmission distances for the four power levels are approximately 4.5 m, 5 m, 5.5 m, and 6 m, respectively. In Plils, every reference node broadcasts data packets including the data fields, its identity, and current power level, periodically with a period of $T_{0}$, by powering in the ascent order. In this setting of Plils, the size of data packets is fixed to 64 bytes, which is adequate for transmitting two critical fields, i.e., the tag’s identity and its current power level. The transmission rate is 1 Mbps, and the receiver sensitivity is −85 dBm [14]. By considering the fact that the transmission delay between adjacent power levels for this chip is 200 μs, and the propagation time of data packets is negligible in our scenario, the transmission time interval between adjacent data packets, $T_{0}$, is set to 4 ms. Target nodes will receive and process these broadcast packets from reference nodes. At a regular time interval $h_{0}$, according to the received packets, target nodes will form one specific data frame and send it to the reader for positioning themselves. In order to obtain the MRPLs of all reference nodes, target nodes should receive a sufficient amount of broadcast packets from reference nodes during the period of time $h_{0}$, which implies that the value of $h_{0}$ is required to be much greater than that of $T_{0}$. After the reader receives the data frames from target nodes, and then through implementing the localization algorithm, it reports the location estimation of tracking target nodes to specified users.

From the viewpoint of data flow direction, the reader only receives data packets from target nodes, and reference nodes broadcast data packets to target nodes. Target nodes receive data packets sent by reference nodes. Every reference node broadcasts data packets periodically with a period of $T_{0}$, by powering in descending order of power levels. From these received data packets, target nodes can obtain the minimum received power level associated with each reference node. Note that $h_{0}$ must be much larger than $T_{0}$ so that target nodes can collect the MRPLs of all reference nodes within their maximum communication distance. In the setting of Plils, $h_{0}$ is set to 1 s. Within the period of 1 s, since the time interval whereby reference nodes periodically broadcast data packets, $T_{0}$, equals 4 ms, target nodes can receive at most 250 broadcast packets from one reference node. In practical applications, the minimum required amount of received data packets could be reduced significantly, especially for mobile objects with relatively higher movement speeds. In order to get the MRPLs of reference nodes, it is easy to see that the value of $h_{0}$ cannot be less than 4 times the value of $T_{0}$ when reference nodes have four output power levels. Nevertheless, to obtain steady MRPLs, in [13], it has been verified that at least 40 data packets are indispensable to avoid estimation errors caused by accidental errors. Therefore, the minimum value of $h_{0}$ could be set to 40$T_{0}$ = 160 ms. The above example illustrates that the value of $h_{0}$ can be adjusted according to practical localization requirements. Considering the potential impacts of node movements on localization accuracy, the proposed Plils is regarded as a suitable solution for object localization and tracking at lower speed in practical applications, such as patient tracking in a hospital and location detection of assets in a warehouse. After data processing, target nodes form data frames, and send them to the reader.

Without the loss of generality, suppose that there are *n* reference nodes and *m* output power levels supported by wireless chips. We use *s* to denote a given target node, and $p_{i}^{s,j}\left(p_{i}^{s,j}\in \left[1,2,\cdots ,m\right]\right)$ to denote the MRPL of reference node *i* at time $t_{j}$, where i∈[1,2,⋯,n], and $\$j\in N,t_{j}=h_{0}*j$. Note that in this paper, the MRPL of one reference node refers to the minimum transmitted power level so that one target node at a distance can properly receive data frames from the reference node, whose definition is slightly different from that in [9]. The MRPL vector at the current time slot, i.e., the MRPL vector formed by node *s*, can be defined as $f_{s}=\left\{p_{1}^{s,j},p_{2}^{s,j},\cdots ,p_{n}^{s,j}\right\}$. For each time slot $h_{0}$, a data frame $F_{s}^{j}$ is formed in the below format as $F_{s}^{j}$ = <$t_{j},ID_{s},\left(ref_{1},PL_{1}^{s,j}\right),\ldots ,\left(ref_{b},PL_{b}^{s,j}\right),\ldots ,\left(ref_{n},PL_{n}^{s,j}\right)$ >. Here, $t_{j}$ is the current time stamp, $ID_{s}$ is the identity of target node *s*, $ref_{b}$ is the reference node *b*’s ID, and $PL_{b}^{j,i}\left(b\in \left\{1,2,\cdots ,n\right\}\right)$ is the MRPL of reference node *b* at $t_{j}$. $d_{0}$ denotes the distance resolution for movement detection. In our case of nRF24LE1, $d_{0}=0.5$ m. This means that if there is a change on the value of $p_{i}^{s,j}$, this implies that the target node *s* must have moved more than 0.5 m from the previous location. Additionally, the size of data packets formed by target nodes is set to 256 bytes for the case of *n* = 10.

Given a target area, which is divided into $z_{1}\times z_{2}$ square grids of side length $d_{0}$, for accurate localization, first of all a position fingerprint database should be established. For each grid, we take its center point to represent it. In order to facilitate understanding, let *g*, $c_{g}$, $o_{g}$ denote one grid, its corresponding grid center point, and the location of the center point, respectively. During the process of collecting fingerprints, we place one stationery node on the center of each grid to form the radio map of the area. Assume that node *A* is placed at $c_{g}$. Here, the fingerprint of grid *g* is defined as the vector of the MRPLs to all reference nodes $F_{g}=\left\{p_{1}^{A},p_{2}^{A},\cdots ,p_{n}^{A}\right\}$. Node *A* will report vector $F_{g}$ to the reader for all grids, where the radio map of the target area, $F=\{F_{g},g=1,2,\cdots ,\;z_{1},z_{2}\}$, is formed.

The radio map incorporates an obvious feature that nearby grids have similar environments, and therefore they exhibit similar fingerprints. Fingerprint-based localization approaches usually utilize this feature to identify the grid center point, which is closest to the mobile node as the location estimation.

**Figure 2:**
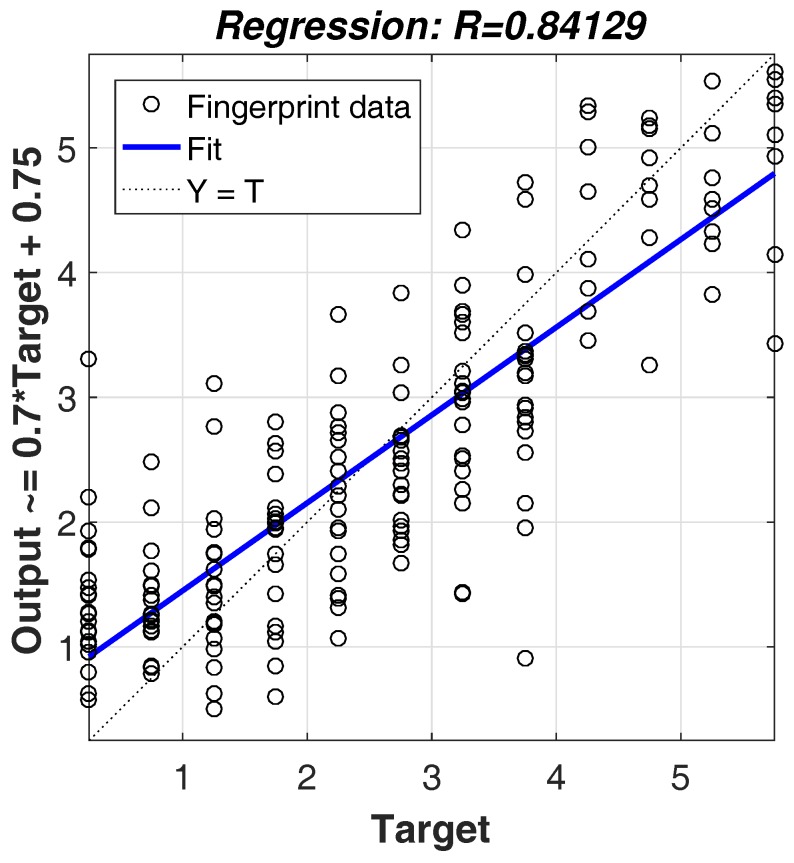
The illustration of the mapping relationship when the back propagation (BP) neural network directly uses plain fingerprint data.

By considering multiple-dimensional fingerprint data that imply the relationship between fingerprint vectors and node locations, the back propagation (BP) neural network method is obviously a suitable option because of its strong ability in complicated multidimensional mapping [15]. However, for BP, directly using a plain fingerprint is infeasible. Due to the impact of obstacles and multipath effects, received signals may vary significantly, resulting in the MRPLs in fingerprint data not obeying a particular radio propagation equation. Therefore, under such complex environments, using one regression model to achieve good mapping from plain fingerprint data to node locations is impossible. For illustrating this observation, we demonstrate in Figure 2 the mapping relationship between plain fingerprint data and node locations, where the value of R represents the mapping degree between fingerprint data and data locations. *R* = 1 means close mapping, and *R* = 0 means a random relationship. From the figure, we have *R* = 0.8419 in our simulation, which means the mapping is not close. In this paper, we introduce subregion clustering to alleviate the issue. By using clustering, subregions whose MRPLs in the corresponding fingerprint data follow a similar radio propagation model will be grouped together. By using BP over each group of subregions, a better mapping degree can be obtained. In addition, for high-dimensional fingerprint data, the empirical results in [16] demonstrated that the 1-NN classifier (as well as the *k*-NN classifier) might lack validity for the similarity mapping problem, even when the data dimensionality is as little as 10. Among all possible classifiers, the SVM classifier has good generalization performance, especially for the case of high-dimensional input space [17]. Hence, the SVM technique is introduced in our solution.

**Figure 3:**
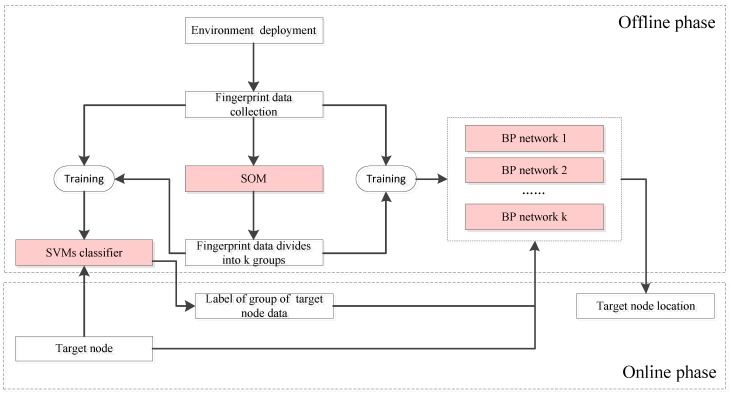
The main framework of the proposed Plils system. SVM: support vector machine; SOM: self-organizing map.

The localization process of Plils consists of two phases; the off-line training phase and the online localization phase. As shown in Figure 3, the whole process adopts three algorithms, i.e., the SOM, SVM, and BP algorithms. Specifically, SOM belongs to an unsupervised learning model, and runs in the offline training phase to categorize the whole radio map into *k* subregions by clustering the fingerprints at all grid center points, while SVM and BP belong to supervised learning models, and span both the off-line training and the online localization phases. SVM regards the *k* subregions obtained by SOM as its training examples, and decides which subregion a target node is located in, while BP utilizes each subregion to train the neural network, and eventually establishes *k* learning models. Let us suppose the subgroup number of the target node is i(i∈[1,k]); the *i*th learning model of BP will be carried out to predict its current location of its target node according to its MRPL vector. Here, *k* is the system parameter and needs to be prespecified in advance. The way to determine the value of *k* is discussed in Section 5.

For the case with a larger localization area, since the transmission range of the used radio modules is limited to 6 m, it is possible that for some locations the target nodes cannot receive data packets from all reference nodes. In order to address this issue, a constant symbol, called not-receive (NR), is introduced. Thus, if two target nodes located in two different grid cells receive data packets from two different sets of reference nodes, the fingerprint data of the two grids should not be categorized into one group in our proposed subregion clustering process. To achieve this goal, we represent the values of NR and four power levels, i.e., −18 dBm, −12 dBm, −6 dBm, and 0 dBm, as 0, 8, 10, 12, and 14, respectively. Obviously, after renumbering, the value differences between NR and the four power levels become larger so that grid cells (where signals from different sets of reference nodes are received) have less of a chance of being categorized into one group by the SOM algorithm. In summary, by introducing the symbol of NR, our proposed localization solutions can be easily extended to scenarios with larger areas.

## 3. The Detailed Implementation Procedure of Plils

### 3.1. The Proposed SOM Algorithm

SOM is a common clustering technique, and is used to group the fingerprint data of the radio map in this paper. The SOM consists of components called neurons, which have the same dimension as fingerprint data. By *k* neurons iteratively recognizing the neighboring section of the fingerprint data, the set of the fingerprint data is partitioned into *k* subsets correspondingly. Here, *k* neurons with locations of dimension 1×n in the layer of the SOM are arranged in a grid topology, denoted by the set $N^{\prime }=\left\{n_{1}^{\prime },n_{2}^{\prime },\cdots ,n_{k}^{\prime }\right\}$, wherein the distance function $D_{i,j}$ between neurons $n_{i}^{\prime }$ and $n_{j}^{\prime }$ (to avoid confusion, we will also use vector $n_{i}^{\prime }=\left[n_{i,1}^{\prime },n_{i,2}^{\prime },\cdots ,n_{i,n}^{\prime }\right]$ to denote the location of neuron $n_{i}^{\prime }$) is defined as

(1) $$D_{n_{i}^{\prime },n_{j}^{\prime }}=\sqrt{\sum_{q=1}^{n}{\left|\left(n_{i,q}^{\prime }-n_{j,q}^{\prime }\right)\right|}^{2}}$$

The detailed implementation process is depicted as below.

(1) First the locations of *k* neurons are randomly set by initializing each element of their location vectors with a random value ranging from 1 to *m*. Note that *m* is the number of power levels supplied by wireless nodes. Other system parameters, including the maximum number of iterations $T^{\prime }$, the radius $d^{\prime }$ of neighboring neurons, and the current iteration time $t^{\prime }$ are initialized to 1.
(2) Consider the fingerprint data set of the target area $F=\{F_{g},g=1,2,\cdots ,\;z_{1},z_{2}\}$. We choose one fingerprint vector $F_{g}$ from F. Then according to Equation (1), we calculate the distances between $F_{g}$ and all neurons $n_{1}^{\prime }$, $n_{2}^{\prime }$, ⋯, $n_{k}^{\prime }$. After that, the neuron with the smallest distance, e.g., $n_{j}^{\prime }$ is selected as a winning neuron of $F_{g}$. Denoting the set of all neurons within $d^{\prime }$ radius of $n_{j}^{\prime }$, including $n_{j}^{\prime }$, as $N_{j}^{\prime }\left(d^{\prime }\right)$, the locations of these neurons are updated by
  (2) $$n_{q}^{\prime }=n_{q}^{\prime }+\eta \left(t^{\prime }\right)h\left(d^{\prime },t^{\prime }\right)\left(F_{g}-n_{q}^{\prime }\right)\;\;n_{q}^{\prime }\in N_{j}^{\prime }\left(d^{\prime }\right)$$
  In Equation (2), $\eta \left(t^{\prime }\right)\left(0<\eta \left(t^{\prime }\right)<1\right)$ is a gain function and monotonically decreases with time $t^{\prime }$. $h(d^{\prime },t^{\prime })$ denotes a neighborhood function. The product of $\eta \left(t^{\prime }\right)h\left(d,t^{\prime }\right)$ is applied in a commonly used smoothing process. In this paper, $\eta \left(t^{\prime }\right)=1-\frac{t^{\prime }}{T^{\prime }}$, and $h\left(d^{\prime },t^{\prime }\right)=exp\left(-\frac{\left|\right|F_{g}-n_{q}^{\prime }{\left|\right|}^{2}}{2\sigma^{2}\left(t^{\prime }\right)}\right)=exp\left(-\frac{D_{F_{g},n_{q}^{\prime }}}{2\sigma^{2}\left(t^{\prime }\right)}\right)$. $\sigma (t^{\prime })$ is also a monotonically increasing function of $t^{\prime }$. Its value is required to be very large when $t^{\prime }$ = 1, and gradually decrease with the increase of $t^{\prime }$. It becomes a fraction of the original value when $t^{\prime }$ increases to 1000. In this paper, we define $\sigma \left(t^{\prime }\right)=-4ln\frac{t^{\prime }}{T^{\prime }}+5$, which coincides with the above requirements. Let $t^{\prime }=t^{\prime }+1$.
(3) Repeat step 2, until $t^{\prime }$ has reached the maximum number of iterations $T^{\prime }$.
(4) Through the finally obtained locations of *k* neurons, all neurons can calculate the distance between all fingerprint vectors and themselves. For any fingerprint vector $F_{g}$, by comparing the distance between itself and every neuron, supposing that the distance of $n_{i}^{\prime }$ is the minimum, then the fingerprint vector $f_{g}$ will be assigned to fingerprint subset ${F^{\prime }}^{i}$.
(5) If all fingerprint vectors in set G have been assigned, the obtained subsets ${F^{\prime }}^{1}$, ${F^{\prime }}^{2}$, ⋯, and ${F^{\prime }}^{k}$ are the clusters formed by the SOM. It is easy to see that $G={F^{\prime }}^{1}\cup {F^{\prime }}^{2}\cup \cdots \cup {F^{\prime }}^{k}$. It implies that the whole target regions can be partitioned into *k* subregions $\left\{F^{i},i=\left[1,2,3,\cdots ,k\right]\right\}$.

### 3.2. SVM and BP

Consider that nearby nodes within one small grid cell have similar environments. Hence, they will receive similar MRPLs from same reference nodes, which will result in the corresponding fingerprint vectors of these nodes being the same or very similar to each other. We leverage this characteristic. In this paper, the SVM is used to identify, among *k* subregions, which subregion a unknown node is located in based on its MRPL vector from the reference nodes, which belongs to a multiclass classification problem. For simplicity, the most popular SVM algorithm, called LibSVM [18], is adopted. It needs an offline training phase to train its learning model. During this phase, all fingerprint vectors in F and their corresponding subset indices are inputs to the LibSVM, where fingerprint vectors are example data, and their subset indices are training labels. In the online localization phase, the MRPL vector of one target node is roughly treated as the fingerprint vector of its nearby grid center point. According to the MRPL, LibSVM harnesses the learning model established to obtain the corresponding index of the subregion that the target node is located in.

Similar to SVM, the learning model of the BP neural network is required to construct in the off-line training phase as well. Here, BP is used to pinpoint the coordinate of the target node. According to the subregion index i(i∈[1,k]) obtained by LibSVM, the *i*th BP network is invoked, and further presents the coordinate of the node within the possible region of radius λ centered at the last calculated localization via the MRPL vector of the target node. From the context, it is easy to see that there exist *k* BP networks. The *i*th BP network will take $F^{\prime }$ as its training data, and their corresponding locations as its training label, to adjust the network structure, setting up its learning model. Specifically, we used the BP algorithm adopted by MATLAB tools [19], the Levenberg–Marquardt back propagation algorithm, to train and classify, namely TrainLM. The corresponding TrainLM network utilizes the received minimum power level vector to get the location of some center point as its output, and the location is roughly regarded as the location of the target node.

The pseudo code of the whole process is depicted as Algorithm 1.

**Algorithm 1:** The implementation process of Plils.
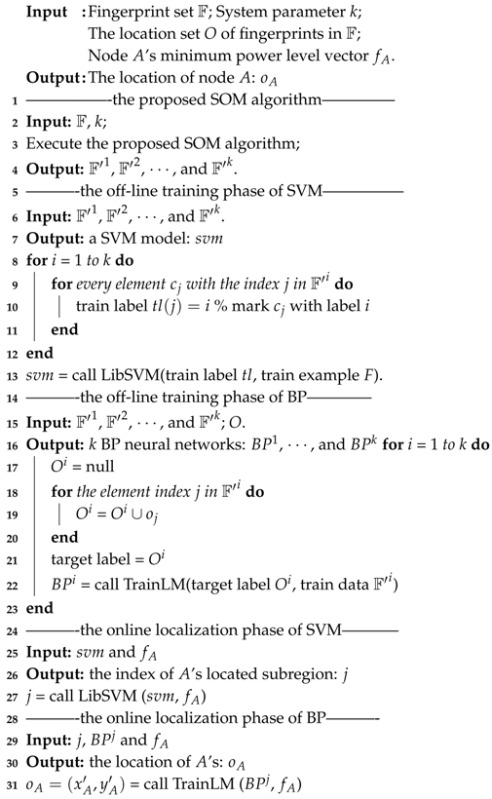


## 4. Experimental Validation

**Figure 4:**
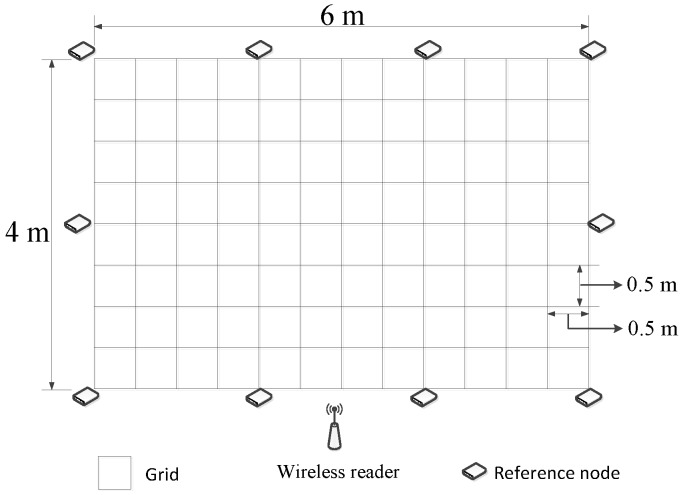
An illustrated layout of the experimental environment.

In order to evaluate the performance of our proposed Plils system, we implemented a prototype system with 10 reference nodes, which were deployed on the perimeter of a 6 m × 4 m rectangular area. All adjacent nodes were spaced by 2 m. The target region of 6 m × 4 m was divided into 96 square grids due to $d_{0}=0.5$ in our system using nRF24LE1, as illustrated in Figure 4. The real deployment of Plils is shown in Figure 1, where chairs were used as obstacles to validate the robustness of PLils with respect to indoor obstacles. We employed CMTL+ and BP as benchmarks to evaluate the superiority of our proposed algorithm in the same experiment environment. All localization algorithms were performed on an external computer connected to the reader. In the offline training phase, according to [13], up to 60 data samples at each grid center point were adequate to obtain steady fingerprint data of the grid. In the online localization phase, the current localization of the target node was estimated based on the data frame (containing MRPL information) reported by the target node in the time slot $h_{0}$. In order to facilitate calculating the localization accuracy, we place a target node at randomly-chosen grid center points. We randomly selected 40 grid center points as testing locations, while their actual locations were recorded. For each testing location, we collected 400 data frames for each measurement. All results were averaged over 20 runs. We considered three cases with respect to the ranges of regions that the target node might be located in, i.e., a small range with $\lambda =d_{0}$, a median range with $\lambda =2d_{0}$, and a comparatively large one with $\lambda =3d_{0}$. To measure the localization accuracy, the localization error distance *e* is used as the performance metric, which is defined as the Euclidean distance between the actual location $\left(x_{r},y_{r}\right)$ and the estimated location $\left(x^{\prime },y^{\prime }\right)$, given by

(3) $$e=\sqrt{{\left(x_{r}-x^{\prime }\right)}^{2}+{\left(y_{r}-y^{\prime }\right)}^{2}}$$

### 4.1. Effect of the Number of Subregions

For the SOM algorithm, an optimal value of *k* needs to be found. According to the experimental investigations from Table 2, when λ=1, with the increase of *k*, the average value of the localization error distance *e* gradually decreases. When k=4, the best localization accuracy can be achieved. For the case of k≥5, the localization accuracy begins to degrade. When λ=2, k=4 is also the best option. When λ=3, although the best localization accuracy occurs at k=5, the obtained localization accuracy when k=4 approaches that when k=5. Thus, it can be seen that in most cases, k=4 is the best choice. In the following experiments, we set k=4. In future work, we will derive the mathematical relationship between *k*, λ and the localization accuracy, so as to calculate the optimal value of *k*.

### 4.2. Comparisons of Algorithms on Localization Accuracy

In order to highlight the effect of the proposed SOM algorithm, three algorithms were tested. They are the BP, SOM + SVMBP, and CMTL+ algorithms. In contrast to SOM + SVMBP, the BP algorithm directly uses the plain fingerprint data for localization. We demonstrate the achieved localization error distance for the three algorithms in Table 3. From the table, it can be observed that, for the CMTL+ algorithm, there is one average localization error distance, while both BP and SOM + SVMBP algorithms have three such distances for three different values of λ, where λ means the radius of possible location regions of the target node. Since the CMTL+ algorithm does not take into account the relationship between the target node’s current location and its possible region after one time slot, only one average localization error distance is obtained. It can be also observed that CMTL+ has the worst performance in terms of localization accuracy. This is because there always exist obstacles in the real deployment environment. In CMTL+, it is assumed that any intermediate location point between two given terminal points must have a fingerprint, which falls in the interval range between the fingerprints tied to the two terminal points. However, in real environments with large obstacles, the actual fingerprints at virtual grid points are often different from the calculated fingerprints corresponding to virtual reference nodes. For instance, in our experiments, a large portion of fingerprints up to 15% have a distance difference of more than 1 between the actual and the calculated values. In addition, compared to BP, SOM + SVMBP can localize target nodes more accurately, with an improvement in localization accuracy from 4.7% to 11.8%. This clearly demonstrates the benefits of the introduction of SOM algorithm. In the best case, SOM + SVMBP achieves an localization accuracy of 75 cm on average.

**Figure 5:**
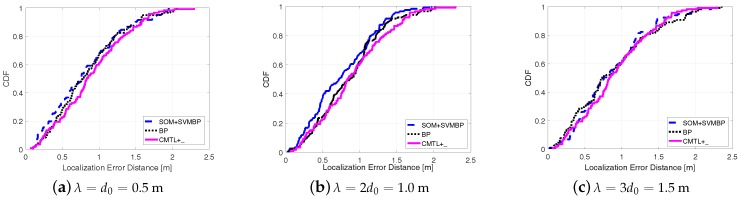
Cumulative distribution function (CDF) of localization error distance for different λ under environments with obstacles.

**Figure 6:**
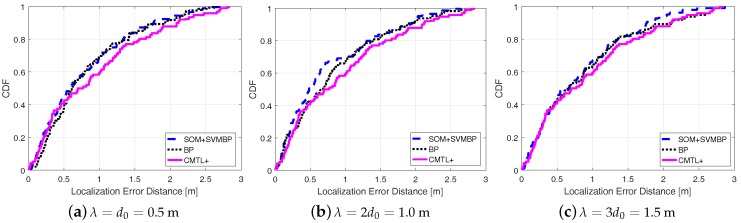
Cumulative distribution function (CDF) of localization error distance for different λ under empty environments.

**Figure 7:**
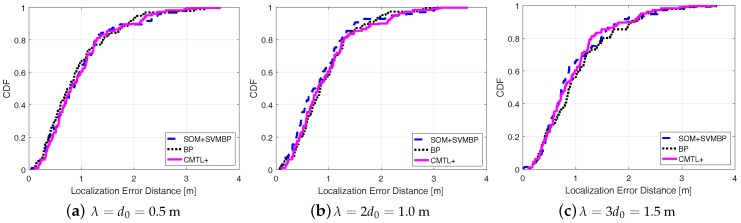
Cumulative distribution function (CDF) of localization error distance for different λ under obstacle-rich environments.

Figure 5 shows the comparisons among three algorithms in terms of the cumulative localization error distance. From the figure, for all cases of λ, we can see that SOM + SVMBP always performs best, whereas CMTL+ performs the worst. Specifically, when λ=1, for SOM + SVMBP, the 50th percentile has an error distance of around 78 cm, and for the 90th percentile it is around 151 cm, while for CMTL+ these two values are 82 cm and 162 cm, respectively. In order to evaluate the robustness to indoor obstacles, we also perform the comparison experiments under two different environments: an empty room and an obstacle-rich lab, wherein there are 10 scattering thick wood desks. Figure 6 and Figure 7 present the experimental results on the achieved localization error distances under empty and obstacle-rich environments, respectively. In these figures, we can observe that for CMTL+, the localization error distances obviously vary under the three different environments. Nevertheless, for SOM + SVMBP, there is no prominent change of the localization error distances. For all these environments, in general, the fingerprinting-based localization strategy can produce a unique fingerprint vector for each sample location. Therefore, SOM + SVMBP is an inherently robust localization algorithm. The results demonstrate the robustness of the proposed Plils to obstacles.

## 5. Conclusions

In this paper, a power level-based wireless indoor localization system, Plils, was proposed. This prototype system was built on a type of COTS wireless chip, nRF24LE1, which supplies four discrete power levels. The localization procedure employs the idea of a fingerprint. A systematic framework was designed firstly. Specifically, it consisted of two phases: an offline training phase and an online localization phase. In contrast to conventional ILS approaches such as LANDMARC and CMTL+, a SOM algorithm suitable to the system was proposed to divide the constructed radio map of the target region into several subregions, which can avoid the BP neural network algorithm (which causes large localization error in the later process). The proposed Plils system showed that the cost-effective system built using the nRF24LE1 chip achieves a median localization accuracy of less than 1 m. Its superiority is shown on comparison with the clustering-based work, CMTL+.

## Figures and Tables

**Table 1:** Rough prices for several popular wireless communication chips.

nRF24LE1	CC2530	LSR450	CC2540
$0.5∼1	$1.5∼2	$5∼10	$2∼5

**Table 2:** The localization error distance (cm) of the SOM + SVMBP of the parameters.

	*k* = 2	*k* = 3	*k* = 4	*k* = 5
$\lambda =d_{0}=0.5$ m	83.12	82.03	80.11	82.96
$\lambda =2d_{0}=1.0$ m	81.10	77.65	74.68	78.41
$\lambda =3d_{0}=1.5$ m	89.42	85.88	83.12	83.02

**Table 3:** The localization error distance (cm) for different algorithms.

	BP	SOM + SVMBP	CMTL+
$\lambda =d_{0}=0.5$ m	82.98	80.11	-
$\lambda =2d_{0}=1$ m	84.73	74.68	89.59
$\lambda =3d_{0}=1.5$ m	87.25	83.12	-
